# Supplementary material for: Comparing the Therapeutic Efficacies of Lung Cancer: Network Meta-Analysis Approaches
Source: Int J Environ Res Public Health. 2022 Nov 2;19(21):14324. doi: 10.3390/ijerph192114324 (PMC9657796; doi:10.3390/ijerph192114324)
Supplement: Supplementary file 1 [file ijerph-19-14324-s001.zip › ijerph-1959202-supplementary.pdf]

Table S1. Papers included in the updated meta-analysis

No	Paper (Year)	Title
1	Rittmeyer A et al (2016)	Atezolizumab versus docetaxel in patients with previously treated non-small-cell lung cancer (OAK): a phase 3, open-label, multicentre randomised controlled trial
2	Wu Y-Q et al (2011)	The Clinical Investigation of Pemetrexed Plus Carboplatin as an Active and Tolerable Treatment Plan in Chinese Elderly Patients with Advanced Lung Adenocarcinoma
3	Goss G.D. et al (2013)	Gefitinib Versus Placebo in Completely Resected Non–Small-Cell Lung Cancer: Results of the NCIC CTG BR19 Study
4	Kelly K. et al (2015)	Adjuvant Erlotinib Versus Placebo in Patients with Stage IB-IIIA Non–Small-Cell Lung Cancer (RADIANT): A Randomized, Double-Blind, Phase III Trial
5	Luo X.-H. et al (2014)	Effect of Erlotinib as Second-Line Therapy on Advanced Non-Small Cell Lung Cancer Following Failure of Chemotherapy
6	Chen Y.-M. et al (2015)	Erlotinib or Chemotherapy in Second-Line or Later Treatment of Tumor EGFR Wild-Type Pulmonary Adenocarcinoma Patients
7	Gao J.-W. et at (2017)	Erlotinib-based doublet targeted therapy versus erlotinib alone in previously treated advanced non-small-cell lung cancer: a meta-analysis from 24 randomized controlled trials
8	Zhuansun Y. et al (2017)	Anti-PD-1/PD-L1 antibody versus conventional chemotherapy for previously-treated, advanced non-small-cell lung cancer: a metaanalysis of randomized controlled trials
9	Hida T. et al (2017)	Atezolizumab in Japanese Patients With Previously Treated Advanced NoneSmall-Cell Lung Cancer: A Subgroup Analysis of the Phase 3 OAK Study
10	Fehrenbacher L. et al. (2016)	Atezolizumab versus docetaxel for patients with previously treated non-small-cell lung cancer (POPLAR): a multicentre, open-label, phase 2 randomised controlled trial
11	Wu D.-P. et al	Phase II trial of gefitinib in female patients with

	(2011)	previously treated advanced non-small cell lung cancer
12	Cheng H.-Y. et al (2010)	Clinical effect of continuous intravenous drip low- dose paclitaxel plus cisplatin treatment for advanced non-small- cell lung cancer
13	Liang Y. et al (2010)	Randomized trial of weekly paclitaxel and cisplatin versus paclitaxel and cisplatin every three weeks in the treatment of elderly advanced NSCLC
14	Xiong Y. et al (2010)	Clinical analysis of efficacy in docetaxel plus cisplatin chemotherapy with 3-DCRT treating the patients with locally advanced NSCLC
15	Liu C.-F. et al (2018)	Efficacy of Erlotinib in Post-Chemotherapy EGFR Wild-Type Lung Adenocarcinoma Patients
16	Xing PY et al (2013)	Efficacy and Safety of Albumin-bound Paclitaxel in Treating Recurrent Advanced Non-small Cell Lung Cancer
17	He Z.-R. et al (2014)	Study of Three-Dimentional Conformal Radiotherapy Combined with Concurrent Pemetrexed Chemotherapy and ErIotinib Maintenance in Treatment of Non-Squamous Cell Carcinoma of Locally Advanced Lung Cancer
18	Scagliotti G. V. et al (2011)	Randomized Phase III Study of Surgery Alone or Surgery Plus Preoperative Cisplatin and Gemcitabine in Stages IB to IIIA Non–Small-Cell Lung Cancer
19	Mattson K.V. et al (2003)	Docetaxel as neoadjuvant therapy for radically treatable stage III non-small-cell lung cancer: a multinational randomized phase III study
20	Felip E. et al (2010)	Preoperative Chemotherapy Plus Surgery Versus Surgery Plus Adjuvant Chemotherapy Versus Surgery Alone in Early-Stage Non–Small-Cell Lung Cancer
21	Pisters K.M.W. et al (2010)	Surgery With or Without Preoperative Paclitaxel and Carboplatin in Early-Stage Non–Small-Cell Lung Cancer: Southwest Oncology Group Trial S9900, an Intergroup, Randomized, Phase III Trial
22	Chang J.Y. et al (2015)	Stereotactic ablative radiotherapy versus lobectomy for operable stage I non-small-cell lung cancer: a pooled analysis of two randomised trials
23	Port J.L.et al (2014)	A Propensity-Matched Analysis of Wedge Resection and Stereotactic Body Radiotherapy for Early Stage Lung

		Cancer
24	Berg LLVD et al (2015)	Patterns of Recurrence and Survival after Surgery or Stereotactic Radiotherapy for Early Stage NSCLC
25	Hamaji M. et al (2015)	Video-Assisted Thoracoscopic Lobectomy Versus Stereotactic Radiotherapy for Stage I Lung Cancer
26	Puri V. et al (2015)	Treatment Outcomes in Stage I Lung Cancer *A Comparison of Surgery and Stereotactic Body Radiation Therapy*
27	Ready N. et al (2018)	Third-Line Nivolumab Monotherapy in Recurrent SCLC: CheckMate 032
28	Maemondo M et al (2010)	Gefitinib or Chemotherapy for Non–Small-Cell Lung Cancer with Mutated EGFR
29	Vyfhuis M.A.L. et al (2017)	Oncological outcomes from trimodality therapy receiving definitive doses of neoadjuvant chemoradiation (≥60 Gy) and factors influencing consideration for surgery in stage III non-small cell lung cancer
30	Reck M. et al(2016)	Pembrolizumab versus Chemotherapy for PD-L1–Positive Non–Small-Cell Lung Cancer
31	Kim Y.J. et al (2018)	Efficacy and Safety of Afatinib for *EGFR*-mutant Non-small Cell Lung Cancer, Compared with Gefitinib or Erlotinib
32	Sun S. et al (2017)	Exploratory cohort study and meta-analysis of *BIM* deletion polymorphism in patients with epidermal growth factor receptor-mutant non-small-cell lung cancer treated with epidermal growth factor receptor tyrosine kinase inhibitors
33	Herbst R.S. et al (2016)	Pembrolizumab versus docetaxel for previously treated, PD-L1-positive, advanced non-small-cell lung cancer (KEYNOTE-010): a randomised controlled trial
34	Torigoe H. et al (2017)	Induction chemoradiotherapy using docetaxel and cisplatin with definitive-dose radiation followed by surgery for locally advanced non-small cell lung cancer
35	Yang J.J. et al (2017)	A phase III randomised controlled trial of erlotinib vs gefitinib in advanced non-small cell lung cancer with EGFR mutations
36	Sequist L.V. et al (2013)	Phase III Study of Afatinib or Cisplatin Plus Pemetrexed in Patients with Metastatic Lung Adenocarcinoma with *EGFR* Mutations

37	Zhou C.C. et al (2011)	Erlotinib versus chemotherapy as first-line treatment for patients with advanced *EGFR* mutation-positive non-small-cell lung cancer (OPTIMAL, CTONG-0802): a multicentre, open-label, randomised, phase 3 study
38	Han J.-Y. et al (2012)	First-SIGNAL: First-Line Single-Agent Iressa Versus Gemcitabine and Cisplatin Trial in Never-Smokers With Adenocarcinoma of the Lung
39	Mok T. S. et al (2009)	Gefitinib or Carboplatin–Paclitaxel in Pulmonary Adenocarcinoma
40	Karim N. A. et al (2014)	Phase II Clinical Trial of Gefitinib for the Treatment of Chemonaïve Patients with Advanced Non-small Cell Lung Cancer with Poor Performance Status
41	Ma L. et al (2017)	Survival and prognostic factors of non-small cell lung cancer patients with postoperative locoregional recurrence treated with radical radiotherapy
42	Shiroyama T. et al (2017)	Pretreatment advanced lung cancer inflammation index (ALI) for predicting early progression in nivolumab-treated patients with advanced non–small cell lung cancer
43	Blackhall F. et al (2017)	Final results of the large-scale multinational trial PROFILE 1005: efficacy and safety of crizotinib in previously treated patients with advanced/metastatic ALK-positive nonsmall-cell lung cancer
44	Lin Y.-T. et al (2018)	Real-World Crizotinib Use for Anaplastic Lymphoma Kinase (ALK)-Positive Advanced Non-Small Cell Lung Cancer under First-Year National Health Insurance Coverage in Taiwan
45	Peters S. et al (2017)	Phase II Trial of Atezolizumab As First-Line or Subsequent Therapy for Patients With Programmed Death-Ligand 1–Selected Advanced Non–Small-Cell Lung Cancer (BIRCH)
46	Gandhi L. et al (2018)	Pembrolizumab plus Chemotherapy in Metastatic Non–Small-Cell Lung Cancer
47	Antonia S.J. et al (2017)	Durvalumab after Chemoradiotherapy in Stage III Non–Small-Cell Lung Cancer
48	Senan S. et al (2016)	PROCLAIM: Randomized Phase III Trial of Pemetrexed-Cisplatin or Etoposide-Cisplatin Plus Thoracic Radiation Therapy Followed by Consolidation

		Chemotherapy in Locally Advanced Nonsquamous Non–Small-Cell Lung Cancer
49	Santiesteban E. et al (2014)	Safety and Efficacy of Racotumomab-Alum Vaccine as Second-Line Therapy for Advanced Non-Small Cell Lung Cancer
50	Alfonso S. et al (2014)	A Randomized, Multicenter, Placebo-Controlled Clinical Trial of Racotumomab-Alum Vaccine as Switch Maintenance Therapy in Advanced Non–Small Cell Lung Cancer Patients
51	Sum Y. et al (2013)	Long-term results of a randomized, double-blind, andplacebo-controlled phase III trial: Endostar (rh-endostatin) versus placebo in combination with vinorelbine and cisplatin in advanced non-small cell lung cancer
52	Guo J.-C. et al (2018)	Efficacy and safety of Endostar combined with vinorelbine and cisplatin for the treatment of advanced non-small cell lung cancer: a comparative study
53	Zhou S.Y. et al (2018)	Efficacy and safety of rh-endostatin (Endostar) combined with pemetrexed/cisplatin followed by rh-endostatin plus pemetrexed maintenance in non-small cell lung cancer: A retrospective comparison with standard chemotherapy
54	Nakajima K. et al (2018)	Clinical outcomes of image-guided proton therapy for histologically confirmed stage I non-small cell lung cancer
55	Lesueur P. et al (2018)	Safety of combined PD-1 pathway inhibition and radiation therapy for non-small-cell lung cancer: A multicentric retrospective study from the GFPC
56	Ross H.J. et al (2006)	A randomized, multicenter study to determine the safety and efficacy of the immunoconjugate SGN-15 plus docetaxel for the treatment of non-small cell lung carcinoma
57	Garassino M. C. et al (2013)	lung cancer: A multicentric retrospective study from the GFPC<br>Erlotinib versus docetaxel as second-line treatment of patients with advanced non-small-cell lung cancer and wild-type *EGFR* tumours (TAILOR): a randomised controlled trial
58	Hanna N. et al	Randomized Phase III Trial of Pemetrexed Versus

	(2004)	Docetaxel in Patients With Non–Small-Cell Lung Cancer Previously Treated With Chemotherapy
59	Ksienski D. et al (2018)	Efficacy of Nivolumab and Pembrolizumab in Patients with Advanced Non-Small-Cell Lung Cancer Needing Treatment Interruption Because of Adverse Events: A Retrospective Multicenter Analysis
60	Karampeazis A. et al (2013)	Pemetrexed versus Erlotinib in Pretreated Patients with Advanced Non–Small Cell Lung Cancer: A Hellenic Oncology Research Group (HORG) Randomized Phase 3 Study
61	Zhang Z.-Q. et al (2016)	Effects of DC,CIK Combined with Conventional Chemotherapy on Prolonging Survival, Improving Quality of Life of Patients with Advanced NSCLC
62	Fu J.-P. et al (2016)	Evaluation of efficiency of induction and consolidation chemotherapy for stage III NSCLC
63	Sun Y.L. et al (2016)	Efficacy and Safetv of Pemetrexed First-1ine Continued Consolidation in Pemetrexed/Carboplatin Chemotherapy for Elderly Patients with Advanced NSCLC
64	Lyu J.H. et al (2015)	A Prospective Phase II Study of Consolidation Chemotherapy after Concurrent Chemoradiotherapy for Oligometastatic Stage IV Non-small Cell Lung Cancer
65	Zhao Y.N. et al (2017)	Efficacy and Safety of Lobaplatin Plus Vinorelbine in Postoperative Adjuvant，Chemotherapy for Patients with NSCLC
66	Gong Z. et al (2017)	Aiyu Capsules or Fufang Banmao Capsules combined with icotinib hydrochloride in the treatment of advanced NSCLC
67	Zhou Y. (2017)	Observation of clinical efficacy and its influence on quality of life of pemetrexed combined with nadaplatin for advanced NSCLC
68	Qi Y.-J. (2012)	Effect of Shenqi Fuzheng Injection Oil The Quality of Life in Patients with Lung Cancer Receiving Chemotherapy after Undergoing Surgery
69	Zhou C.-H. et al (2008)	Clinical evaluation of Shenqi Fuzheng injection in the chemo-radiotherapy in treatment of non-small-cell lung cancer
70	Wang Y.-X. et al (2009)	Shenqi Fuzheng injection combined with three dimensional-conformal radiotherapy for old patient's

		advanced non-small cell lung cancer
71	Liu C.-L. et al (2004)	Effect of Shenqi Fuzheng Injection for Assistance of Chemotherapy in Treating Senile patients with Non-small Cell Lung Cancer
72	Li Q. et al (2017)	Effects of Shenqi Fuzheng injection combined with chemotherapy on immune function and tumor markers in elderly patients with non-small cell lung cancer
73	Wang W.-M. et al (2011)	The treatment of Shenqifuzheng injection combined with docetaxel and cisplatin for advanced lung cancer
74	Wang Y.Q. et al (2010)	Clinical Observation of Shenqi Fuzheng Injection Combined with Chemotherapy in the Treatment of Advanced Lung Cancer
75	Wang L.Y. et al (2009)	Clinical Observation of Shenqi Fuzheng Injection Combined with Chemotherapy in the Treatment of Advanced Non-small Cell Lung Cancer
76	Basu S. et al (2006)	A prospective and randomized study of radiotherapy, sequential chemotherapy radiotherapy and concomitant chemotherapy-radiotherapy in unresectable non small cell carcinoma of the lung
77	Atagi S. et al (2005)	Standard Thoracic Radiotherapy with or without Concurrent Daily Low-dose Carboplatin in Elderly Patients with Locally Advanced Non-small Cell Lung Cancer: a Phase III Trial of the Japan Clinical Oncology Group (JCOG9812)
78	Huber R.M. et al (2006)	Simultaneous Chemoradiotherapy Compared With Radiotherapy Alone After Induction Chemotherapy in Inoperable Stage IIIA or IIIB Non–Small-Cell Lung Cancer: Study CTRT99/97 by the Bronchial Carcinoma Therapy Group
79	Winton T. et al (2016)	Vinorelbine plus Cisplatin vs. Observation in Resected Non–Small-Cell Lung Cancer
80	Arriagada R. et al (2004)	The International Adjuvant Lung Cancer Trial Collaborative Group. Cisplatin-Based Adjuvant Chemotherapy in Patients with Completely Resected Non–Small-Cell Lung Cancer
81	Rodrigues-Pereira J. et al (2011)	A Randomized Phase 3 Trial Comparing Pemetrexed/Carboplatin and Docetaxel/Carboplatin as First-Line Treatment for Advanced, Nonsquamous Non-

		small Cell Lung Cancer
82	Nawrocki S. et al (2010)	Concurrent Chemotherapy and Short Course Radiotherapy in Patients with Stage IIIA to IIIB Non-small Cell Lung Cancer Not Eligible for Radical Treatment *Results of a Randomized Phase II Study*
83	Scagliotti G.V. et al (2013)	An Open-Label, Multicenter, Randomized, Phase II Study of Pazopanib in Combination with Pemetrexed in First-Line Treatment of Patients with Advanced-Stage Non–Small-Cell Lung Cancer
84	Ferry D. et al (2017)	Carboplatin versus two doses of cisplatin in combination with gemcitabine in the treatment of advanced non-small-cell lung cancer: Results from a British Thoracic Oncology Group randomised phase III trial
85	Tanaka K. et al (2015)	*EGFR* Mutation Impact on Definitive Concurrent Chemoradiation Therapy for Inoperable Stage III Adenocarcinoma
86	Chiappori A. et al (2010)	Phase II, Double-Blinded, Randomized Study of Enzastaurin Plus Pemetrexed as Second-Line Therapy in Patients with Advanced Non-small Cell Lung Cancer
87	Clement-Duchene C. et al (2012)	A phase II study of enzastaurin in combination with erlotinib in patients with previously treated advanced non-small cell lung cancer
88	Foster C.C. et al (2019)	Adjuvant chemotherapy following stereotactic body radiotherapy for early stage non-small-cell lung cancer is associated with lower overall: A National Cancer Database Analysis
89	Perng R.-P. et al (1997)	Gemcitabine Versus the Combination of Cisplatin and Etoposide in Patients with Inoperable Non-Small-Cell Lung Cancer in a Phase II Randomized Study
90	Manegold C. et al (1997)	Single-agent gemcitabine *versus* cisplatin-etoposide: Early results of a randomised phase II study in locally advanced or metastatic non-small-cell lung cancer
91	Sandler A. et al (1999)	Gemcitabine: Single-Agent and Combination Therapy in Non-Small Cell Lung Cancer
92	Le Chevalier P et al (1994)	Randomized study of vinorelbine and cisplatin versus vindesine and cisplatin versus vinorelbine alone in advanced non-small-cell lung cancer: results of a European multicenter trial including 612 patients

93	Laack E. et al (2004)	Randomized Phase III Study of Gemcitabine and Vinorelbine Versus Gemcitabine, Vinorelbine, and Cisplatin in the Treatment of Advanced Non–Small-Cell Lung Cancer: From the German and Swiss Lung Cancer Study Group
94	Zhang Q.Q. et al (2015)	Comparison of single-agent chemotherapy and targeted therapy to first-line treatment in patients aged 80 years and older with advanced non-small-cell lung cancer
95	Cardenal F. et al (1999)	Randomized Phase III Study of Gemcitabine-Cisplatin Versus Etoposide-Cisplatin in the Treatment of Locally Advanced or Metastatic Non–Small-Cell Lung Cancer
96	Stinchcombe T.E. et al (2019)	Effect of Erlotinib Plus Bevacizumab vs Erlotinib Alone on Progression-Free Survival in Patients with Advanced *EGFR*-Mutant Non–Small Cell Lung Cancer A Phase 2 Randomized Clinical Trial
97	Puri V. et al (2012)	A comparison of surgical intervention and stereotactic body radiation therapy for stage I lung cancer in high-risk patients: A decision analysis
98	Wang P. et al (2016)	A propensity-matched analysis of surgery and stereotactic body radiotherapy for early stage non-small cell lung cancer in the elderly
99	Kastelijn E.A. et al (2015)	Clinical Outcomes in Early-stage NSCLC Treated with Stereotactic Body Radiotherapy *Versus* Surgical Resection
100	Sakaguchi M. et al (2016)	Patient outcomes of monotherapy with hypofractionated three-dimensional conformal radiation therapy for stage T2 or T3 non-small cell lung cancer: a retrospective study
101	Crabtree T.D. et al (2013)	Analysis of first recurrence and survival in patients with stage I non–small cell lung cancer treated with surgical resection or stereotactic radiation therapy
102	Verstegen N.E. et al (2013)	Stage I–II non-small-cell lung cancer treated using either stereotactic ablative radiotherapy (SABR) or lobectomy by video-assisted thoracoscopic surgery (VATS): outcomes of a propensity score-matched analysis
103	Stephans K.L.et al (2009)	A Comparison of Two Stereotactic Body Radiation Fractionation Schedules for Medically Inoperable Stage I Non-small Cell Lung Cancer *The Cleveland Clinic*

		Experience
104	Crabtree T. D. et al (2010)	Stereotactic body radiation therapy versus surgical resection for stage I non–small cell lung cancer
105	Grills I.S. et al (2010)	Outcomes After Stereotactic Lung Radiotherapy or Wedge Resection for Stage I Non–Small-Cell Lung Cancer
106	Palma D. et al (2011)	Treatment of stage I NSCLC in elderly patients: A population-based matched-pair comparison of stereotactic radiotherapy versus surgery
107	Varlotto J. et al (2013)	Matched-Pair and Propensity Score Comparisons of Outcomes of Patients with Clinical Stage I Non–Small Cell Lung Cancer Treated with Resection or Stereotactic Radiosurgery
108	Robinson C.G. et al (2013)	Patterns of Failure after Stereotactic Body Radiation Therapy or Lobar Resection for Clinical Stage I Non–Small-Cell Lung Cancer
109	Hamaji M. et al (2015)	Video-Assisted Thoracoscopic Lobectomy Versus Stereotactic Radiotherapy for Stage I Lung Cancer
110	Ren B.C. et al (2015)	Clinical effect study of chemical therapy combined with Endostar treatment on late stage NSCLC patients with the expression of ERCC 1
111	Zhao Z.-Y. et al (2007)	The short-term observation of Shenqi Fuzheng injection combined with NP chemotherapy in treating elderly patients with advanced non-small cell lung cancer
112	Shi X. et al (2007)	Combined treatment of Shenqi Fuzheng injection and chemotherapy on advanced NSCLC
113	Wang S.J. (2009)	Clinical Observation of Shenqi Fuzheng Injection Combined with Chemotherapy in the Treatment of 74 Cases of Senile Small Cell Lung Cancer
114	Wang J.W. et al (2005)	Results of randomized，multicenter，double-blind phase III trial of rh-endostatin(YH-16) in treatment of advanced non-small cell lung cancer patients
115	Le Chevalier T. et al (1994)	Randomized Study of Vinorelbine and Cisplatin Versus Vindesine and Cisplatin Versus Vinorelbine Alone in Advanced Non-Small-Cell Lung Cancer: Results of a European Multicenter Trial Including 612 Patients
